# Data from cyclic tensile tests on sutured organs to evaluate creep behaviour, distraction, and residual thread strength

**DOI:** 10.1016/j.dib.2020.105644

**Published:** 2020-04-30

**Authors:** Giulia Pascoletti, Maria Chiara Pressanto, Giovanni Putame, Mara Terzini, Giordano Franceschini, Elisabetta M. Zanetti

**Affiliations:** a Department of Engineering, University of Perugia, Italy; b Dip. di Medicina Veterinaria – Sezione Chirurgia e Radiodiagnostica, University of Perugia, Italy; c Polito^BIO^MedLab, Politecnico di Torino, Italy; d DIMEAS, Politecnico di Torino, Italy

**Keywords:** Suture testing, Distraction, Failure, Creep, Neuropathy, Tissue relaxation

## Abstract

A number of applications in the surgical practice are based on tensile sutures aimed to keep soft tissues in place and compensate the exit of neuropathies, prolapses or general tissue relaxation. Long-term behaviour of these constructs need to be carefully examined in order to define tensile forces to be applied and to compare different suture anchors.

Data here reported refer to equine laryngoplasties, where a suitable loading system has been designed in order to be able to test sutures in-sito, applying known forces (“On-site testing of sutured organs: an experimental set up to cyclically tighten sutures” (Pascoletti et al., 2020 [1])). The loading protocol was made of two steps: in the first step, 3000 loading cycles have been performed; in the following step, a tensile test up to rupture was performed.

Cyclic load/displacement curves allow evaluating suture distraction, as a consequence of suture migration and/or soft tissues creep. Tensile curves allow evaluating the residual thread strength and its ultimate displacement.

These data can provide a detailed insight of long-term suture behaviour and can be a reference to compare different threads and/or suture anchors.

Specifications table

**Table:** 

Subject	Biomechanics
Specific subject area	Sutures
Type of data	Raw Data in a Table
How data were acquired	Data were acquired performing mechanical tests.
	The loading machine is Instron Electropulse E3000.
	The samples underwent 3000 loading cycles, displacement-controlled (rate equal to 1 mm/s), between 30 N and 50 N force limits.
	Finally, the samples underwent a tensile test up to rupture (displacement rate equal to 1 mm/s).
	The suture was stretched in-situ with known loads, thanks to the system described in a previous work Pascoletti et al. [1].
	Samples were equine laryngoplasties performed by an expert surgeon; they were chosen as representative of prosthetic sutures used to lift soft tissues.
Data format	Raw data concerning 8 samples.
Parameters for data collection	Data have been sampled at 100 Hz
Description of data collection	Data come from the output of the loading machine: force data were measured by a Dynacell biaxial dynamic load cell (axial load range ±5 kN; accuracy equal to 0.10% in the 0–60 N load range here used, according to the most recent calibration curve); displacement was measured by an optical encoder.
Data source location	PolitoBIOMedLab
	Politecnico di Torino
	Torino (ITALY)
	45°03′58.1″N 7°39′30.1″
Data accessibility	Repository name: Mendeley
	Data identification number: doi:10.17632/zpf2jrtgsx.1
	Direct URL to data: https://data.mendeley.com/datasets/zpf2jrtgsx/1
Related research article	Giulia Pascoletti, Maria Chiara Pressanto, Giovanni Putame, Mara Terzini, Alberto L. Audenino, Elisabetta M. Zanetti
	On-site testing of sutured organs: an experimental set up to cyclically tighten sutures
	Journal of Mechanical Behaviour of Biomedical Materials
	In Press

## Value of the data

• These data provide an in-sight into the long-term behaviour of tensile sutures.
• Information here shared can be useful to all surgeons working on soft tissues with localised loads, provided by tensioned sutures: aesthetic surgeons working with thread lifts [2] otorhinolaryngologists performing sling arytenoid adduction [3] or arytenoid abduction lateropexy in neonatal care [4]. Surgeons performing blepharoptosis [5,6], surgeons correcting various kinds of prolapses with sutures [7], and so on.
• These data can be compared to data obtained with different threads or different thread anchorages (suture buttons or suture anchors [8]).
• There is paucity of ‘In vitro’ data concerning suture distraction: most data in literature have been obtained with set up which cannot allow estimating the exact load being carried by the suture, unless the suture alone or the suture constrained on one single end has been tested. This experimental set up allows testing the suture ‘on-site’ so that the compliance and creep behaviour of both its end points is taken into account.

## 1 Data description

This article includes raw data (organised in 16 files and uploaded on Mendeley repository: https://data.mendeley.com/datasets/zpf2jrtgsx/1) and two figures. With reference to raw data, file names have been assigned in this way:

• TestxCyclic.txt: sample *x* (with *x* ranging from 1 to 8), undergoing cyclic loading,
• TestxRamp: sample *x* (with *x* ranging from 1 to 8), undergoing tensile test.

TestxCyclic.txt files are made of 7 columns:

1. Total Time (s)
2. Cycle Elapsed Time (s): time elapsed since the beginning of a new cycle
3. Step: ‘1ʼ when the load is rising; ‘2ʼ when the load is lowering
4. Loop1(1): number of performed cycles
5. Position (mm): absolute position
6. Force (N)
7. Displacement (Linear:Digital Position) (mm): relative displacement, taking zero-load position as a reference

TestxRamp.txt files are made of 5 columns:

1. Total Time (s)
2. Cycle Elapsed Time (s): time elapsed since the beginning of a new cycle
3. Position (mm): absolute position
4. Force (N)
5. Displacement (Linear:Digital Position) (mm): relative displacement, taking zero-load position as a reference.

**Fig. 1:**
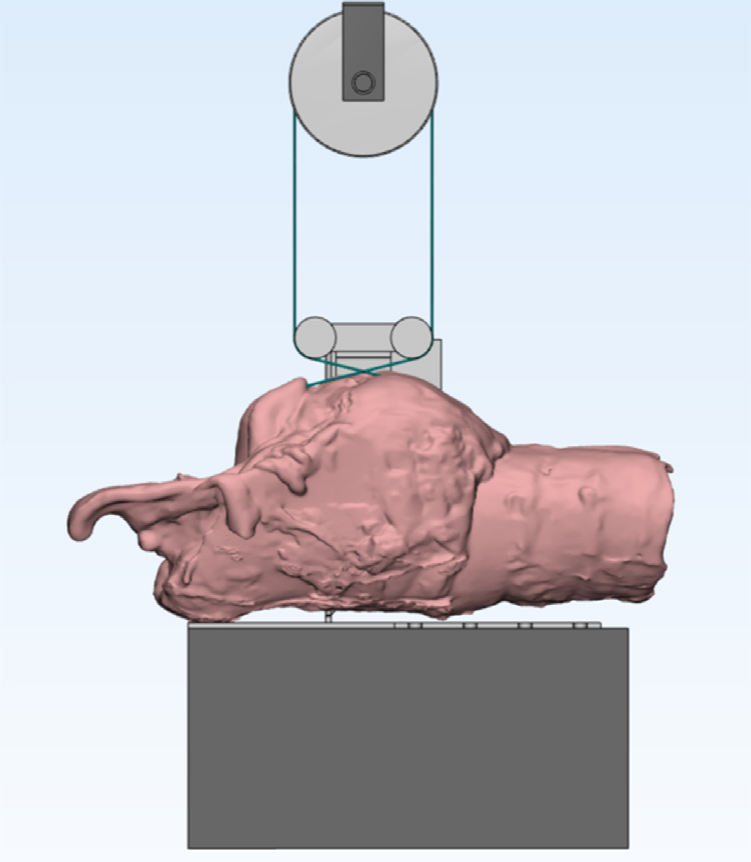
Experimental set-up.

Fig. 1 illustrates the loading set up: the organ is laid on its support; thread ends cross, they pass through lower pulleys and are knotted above an upper pulley, moved by the loading machine.

**Fig. 2:**
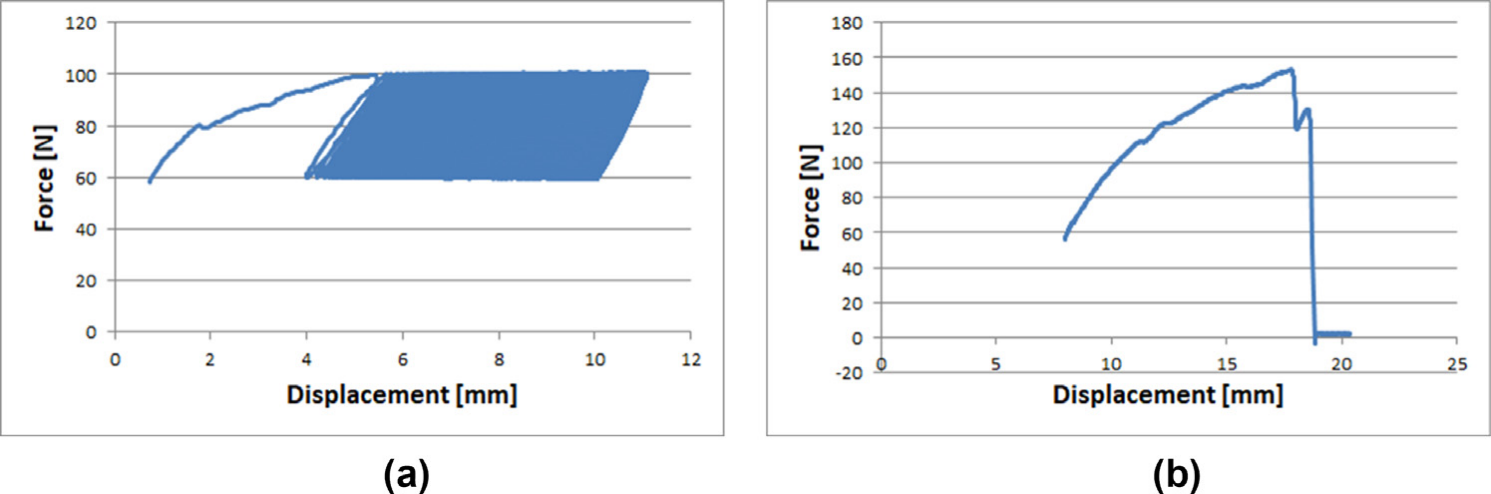
Sample curves obtained during cyclic tests (a) or during the final tensile test (b).

Fig. 2 reports force/displacement patterns for cyclic load (Fig. 2(a)) or for the final load-to-failure test (Fig. 2(b)).

## 2 Experimental design, materials, and methods

The objective of this research has been evaluating the distraction (or creep behaviour) and residual strength of a tensile suture undergoing 3000 cyclic tests.

## 3 Materials

Equine laryngoplasties have been used as a benchmark. This surgery is performed in case of recurrent laryngeal neuropathy and it is aimed to provide enough airflow for the horse. The prosthesis is actually a suture which is placed between the caudal aspect of the cricoid cartilage and the muscular process of the arytenoid cartilage to simulate the action of the cricoarytenoideus dorsalis muscle.

Larynges samples were collected from a local abattoir. They belonged to 5 male and 3 female horses whose age ranged between 3 and 13 years. Laryngoplasties were performed by an experienced veterinary surgeon, who used Ethibond UPS 6 thread.

A suitable experimental set-up has been designed in order to test the suture on-site. It is despicted in Fig. 1.

The organ is securely fastened to the lower base of the loading machine. Lower pulleys allow stressing the suture along its axis, independently from the thread elongation. The load applied by the machine acts on the upper pulley and it is equal to twice the thread tension. Testing the suture on-site allows taking into account the local compliance of both suture ends at the respective anchorage point on the organ.

## 4 Methods

The loading protocol has been so established:

- Step 1: 3000 loading cycles, displacement controlled at 1 mm/s, between two load limits (30 N and 50 N).
- Step 2: tensile test, up to rupture, displacement controlled at 1 mm/s.

The value of load limits and displacement rate have been taken from physiological conditions, according to in vivo measurements [9].

Sample curves have been reported in Fig. 2.
